# Impact of renin–angiotensin system inhibitors discontinuation in advanced chronic kidney disease: a target trial emulation

**DOI:** 10.1093/ckj/sfag304

**Published:** 2026-09-04

**Authors:** Valentin Maisons, Margaux Costes-Albrespic, Sophie Liabeuf, Solène M Laville, Ziad Massy, Angelo Karaboyas, Roberto Pecoits-Filho, Emilie Moutard, Marie Metzger, Jean-Michel Halimi, Etienne Dantan, Natalia Alencar de Pinho, Bénédicte Sautenet

**Affiliations:** Université de Tours, CHU de Tours, Service de Néphrologie, Hôpital Bretonneau, Tours, France; INSERM U1246 - SPHERE (methodS in Patient-centered outcomes and HEalth ResEarch), Université de Tours, Nantes Université, Tours, Nantes, France; Centre for Research in Epidemiology and Population Health (CESP), Paris-Saclay University, Inserm U1018, Versailles Saint-Quentin University, Clinical Epidemiology Team, Villejuif, France; Pharmacoepidemiology Unit, Department of Clinical Pharmacology, Amiens-Picardie University Medical Center, Amiens, France; MP3CV Laboratory, Jules Verne University of Picardie, Amiens, France; Pharmacoepidemiology Unit, Department of Clinical Pharmacology, Amiens-Picardie University Medical Center, Amiens, France; MP3CV Laboratory, Jules Verne University of Picardie, Amiens, France; Centre for Research in Epidemiology and Population Health (CESP), Paris-Saclay University, Inserm U1018, Versailles Saint-Quentin University, Clinical Epidemiology Team, Villejuif, France; AURA (Association pour l’Utilisation du rein Artificiel en Région Parisienne), Paris, France; Arbor Research Collaborative for Health, Ann Arbor, MI, USA; Arbor Research Collaborative for Health, Ann Arbor, MI, USA; Centre for Research in Epidemiology and Population Health (CESP), Paris-Saclay University, Inserm U1018, Versailles Saint-Quentin University, Clinical Epidemiology Team, Villejuif, France; Centre for Research in Epidemiology and Population Health (CESP), Paris-Saclay University, Inserm U1018, Versailles Saint-Quentin University, Clinical Epidemiology Team, Villejuif, France; Université de Tours, CHU de Tours, Service de Néphrologie, Hôpital Bretonneau, Tours, France; INSERM U1246 - SPHERE (methodS in Patient-centered outcomes and HEalth ResEarch), Université de Tours, Nantes Université, Tours, Nantes, France; Centre for Research in Epidemiology and Population Health (CESP), Paris-Saclay University, Inserm U1018, Versailles Saint-Quentin University, Clinical Epidemiology Team, Villejuif, France; Université de Tours, CHU de Tours, Service de Néphrologie, Hôpital Bretonneau, Tours, France; INSERM U1246 - SPHERE (methodS in Patient-centered outcomes and HEalth ResEarch), Université de Tours, Nantes Université, Tours, Nantes, France

**Keywords:** epidemiology, renin–angiotensin system inhibitors, target-trial emulation

## Abstract

**Background:**

Renin–angiotensin system (RAS) inhibitors are a cornerstone of cardiovascular and kidney risk modification in chronic kidney disease (CKD). Yet, they are often discontinued in advanced CKD to limit adverse effects or to delay kidney replacement therapy (KRT).

**Methods:**

From the CKD-Renal Epidemiology and Information Network prospective cohort study, we selected participants with an estimated glomerular filtration rate (eGFR) <30 ml/min/1.73 m^2^ and treated with RAS inhibitors. We performed a target trial emulation with parametric g-formula to compare two strategies: to discontinue or to continue RAS inhibitors. Models were adjusted for sociodemographics, clinical and laboratory characteristics, and drug prescriptions. Outcomes were the composite of cardiovascular death, myocardial infarction, stroke, or hospitalization for heart failure, i.e. major cardiovascular events (MACE), and KRT initiation.

**Results:**

Among the 1434 patients included (median age 68 years, median eGFR 25 ml/min/1.73 m^2^, 35% women), 386 (27%) discontinued RAS inhibitors over a median follow-up of 35 months. Very few events related to adverse drug reactions were reported in the 3 months preceding RAS inhibitor discontinuation (*n* = 23), but their incidence over this period was ∼13-fold higher than that during the entire follow-up. The 3-year adjusted relative risk of MACE associated to discontinuing RAS inhibitors, compared to continuing, was 2.02 (95% Confidence interval (CI), 1.62–2.47), and that of KRT initiation, 1.34 (95% CI, 1.14–1.60).

**Discussion:**

RAS inhibitor discontinuation was strongly associated with MACE and KRT risks, major causes of morbidity, and mortality in advanced CKD. Our findings, consistent with prior data, suggest the risk–benefit balance favors continued use with close monitoring.

## Graphical Abstract

**Graphical Abstract:**
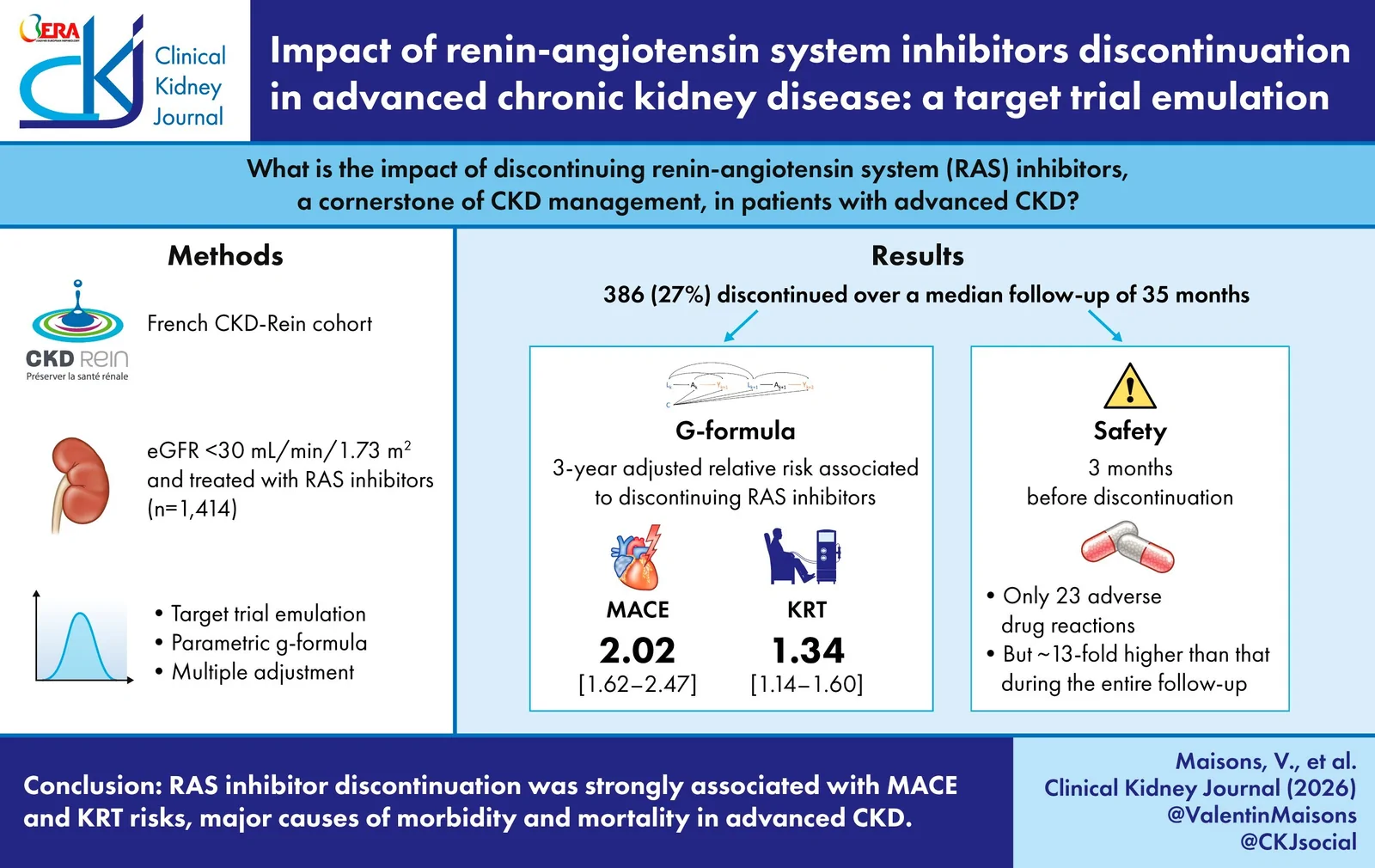

KEY LEARNING POINTS **What was known:**

- Renin–angiotensin system (RAS) inhibitors reduce cardiovascular and kidney risks and are recommended in patients with chronic kidney disease. However, their use in advanced chronic kidney disease (CKD) remains controversial due to concerns about hyperkalemia, acute kidney injury, and declining kidney function.
- In routine practice, RAS inhibitors are frequently discontinued when kidney function declines below an estimated glomerular filtration rate (eGFR) of 30 ml/min/1.73 m², despite limited evidence supporting this strategy.
- Evidence from randomized trials in advanced CKD is scarce, and observational studies have been limited by confounding, leaving uncertainty about the clinical consequences of discontinuing RAS inhibitors.

**This study adds:**

- Using a target trial emulation in the CKD-Renal Epidemiology and Information Network (CKD-REIN) cohort, discontinuation of RAS inhibitors in advanced CKD was associated with a substantially higher risk of major cardiovascular events compared with continuation.
- Discontinuation was also associated with a higher risk of kidney replacement therapy initiation.
- These findings support continued use of RAS inhibitors in advanced CKD when clinically feasible, with careful monitoring for adverse drug reactions.

**Potential impact:**

- These results challenge the common practice of routinely stopping RAS inhibitors in patients with advanced CKD.
- Continuation of RAS inhibitors may reduce cardiovascular events and delay kidney replacement therapy in this high-risk population.
- The findings support guideline recommendations favoring maintenance of RAS inhibitors with close monitoring rather than systematic discontinuation.

## INTRODUCTION

Patients with stages 4 and 5 chronic kidney disease (CKD) are at very high risk of kidney failure and major adverse cardiovascular events (MACE) [1, 2]. The place of renin–angiotensin system (RAS) inhibitors, i.e. angiotensin-converting enzyme inhibitors (ACEi) and angiotensin II receptor blockers (ARBs), as a cornerstone of kidney and cardiovascular risk modification in CKD, or as first-line blood pressure lowering treatment in patients with moderate CKD is consensual in the guidelines [3–6]. On the other hand, the risk–benefit balance of RAS inhibitors in specific populations remains controversial [7]. Despite evidence supporting benefits of their use in patients with CKD stages 4 or 5 [8–12], many reasons exist to discontinue RAS inhibitors in this population. The most noteworthy are the risks of hyperkalemia and acute kidney injury (AKI). Stopping RAS inhibitors has also been considered a way to delay kidney replacement therapy (KRT) initiation, due to its acute effects on estimated glomerular filtration rate (eGFR) [11, 13]. As long as the risk–benefit balance of RAS inhibitors in advanced CKD remains unclear, there may be heterogeneous prescription practices in this population [14, 15].

Clinical decisions about whether to stop or to continue RAS inhibitors in patients with advanced CKD are considerably more common than decisions to initiate these drugs. The multicenter, randomized, controlled Trial of Angiotensin-Converting Enzyme Inhibitor/Angiotensin Receptor Blocker Withdrawal in Advanced Renal Disease (STOP-ACEi) study aimed to assess the difference in eGFR at 3 years across 410 patients with advanced CKD on ACEi or ARBs who were randomized to continue or discontinue RAS inhibitor therapy [8]. In that study, discontinuation of the RAS inhibitor was not associated with a significant difference in eGFR slope between the randomized groups. The trial was underpowered to assess clinical outcomes.

Cardiovascular consequences of RAS inhibitor discontinuation in advanced CKD are of particular concern. Patients with CKD stages 4 and 5 have an exceptionally high cardiovascular risk, and RAS inhibitors may provide cardioprotective effects through blood pressure control and modulation of neurohormonal activation [16, 17]. However, evidence specifically addressing cardiovascular outcomes in this setting remains limited.

We hypothesize that a target trial emulation using observational data [18] would allow us to assess the effect of RAS inhibitor discontinuation with regards to major cardiovascular and kidney events, while considering a broader and more representative population than those usually included in randomized clinical trials, thus capturing real-world effectiveness [19]. Using a target trial emulation approach based on the CKD-Renal Epidemiology and Information Network (CKD-REIN) cohort study, we assessed the effect of RAS inhibitor discontinuation on the risks of MACE and KRT initiation in patients with advanced CKD.

## MATERIALS AND METHODS

### Data source and population

CKD-REIN is a prospective cohort study which enrolled, from July 2013 to April 2016, 3033 adult patients (≥18 years) with a proven diagnosis of CKD stages 3–4 who had neither undergone kidney transplantation nor received chronic dialysis. Patients who moved to CKD stages 2 or 5 between eligibility assessment and inclusion were enrolled, assuming that this would prevent selection bias due to kidney function variability. Recruitment was performed in 40 nationally representative (geographically and from a legal status perspective) outpatient nephrology clinics in France. Complete rationale, design, and methods of the CKD-REIN cohort study have been published previously [20]. The institutional review board of the French National Institute of Health and Medical Research (reference: IRB00003888) approved the protocol, and the study was registered at ClinicalTrials.gov (NCT03381950). All study participants provided informed consent.

CKD-REIN patients were eligible for this analysis if they had an eGFR below 30 ml/min/1.73 m² at inclusion (prevalent) or if they achieved this eGFR threshold during follow-up (incident). In addition, they had to be prescribed a RAS inhibitor for at least 3 months during the observation period.

### Studied characteristics and explanatory variables

Data were collected at baseline and then annually from interviews, medical records, and self-administered questionnaires. They included sociodemographic characteristics, medical history, and laboratory data generated as part of the study protocol or of routine care. Diabetes was defined by the presence of glucose-lowering drug prescription, glycated hemoglobin (HbA1c) ≥6.5%, fasting glucose ≥7 mmol/l, or non-fasting glucose ≥11 mmol/l. Cardiovascular history at baseline included heart failure, coronary artery disease, cerebrovascular disease, and peripheral arterial disease. The urinary albumin-to-creatinine ratio (uACR) was either calculated from original data or estimated from protein-to-creatinine ratio [21]. The eGFR was estimated using the 2009 Chronic Kidney Disease Epidemiology Collaboration (CKD-EPI) creatinine equation [22]. Respiratory disease was defined as any history of chronic lung disease, home oxygen use, or prescription of continuous positive airway pressure. Height and weight, assessed by nephrologists or outpatient nurses, were used to calculate body mass index (BMI, kg/m²). The study protocol required blood pressure (BP) to be measured at least twice, in a seated position, after a 5-min rest; the average of these measurements was used in all analyses. Participants were asked to bring all drug prescriptions (from any doctor) over the 3 months preceding their inclusion visit, and all prescriptions for the preceding year to each annual follow-up visit. Antihypertensive drugs were coded according to the international Anatomical Therapeutic and Chemical (ATC) thesaurus (Table S1) [23].

### Exposure

The main study exposure was the discontinuation of RAS inhibitor prescriptions: ACEi or ARBs. To account for treatment interruptions of short duration (e.g. while awaiting resolution of an episode of dehydration) and to avoid reverse causality (treatment interrupted because of poor health), we considered only interruptions of more than 1 month as discontinuation. In addition, we censored patients at the initiation of dual RAS blockade, i.e. concomitant prescription of ACEi and ARBs. Some patients who had discontinued the drug resumed taking RAS inhibitors during the follow-up (*n* = 119); their status (whether they continued or discontinued) was determined according to the aforementioned conditions, i.e. a minimum of 3 months of RAS inhibitor prescription was required for the “continuation” status, a minimum withdrawal duration of 1 month for the “discontinuation” status. To assess whether RAS inhibitor discontinuation was potentially linked to poor tolerability, we ascertained adverse drug reactions considered relevant for RAS inhibitor risk–benefit assessment—namely, acute kidney injury, hypotension, hyperkalemia occurring within the 3 months immediately preceding the drug discontinuation. Adverse drug reactions over the CKD-REIN study follow-up were identified through medical records, patient interviews with clinical research associates, and hospital reports; information was then reviewed by two pharmacists (S.L. and S.M.L.). The potential causal relationship with the RAS inhibitor was evaluated based on the official procedure used to report adverse drug reactions to the national health authorities in France, as previously reported [24].

### Studied outcomes

All hospitalizations occurring during study follow-up were identified from medical reports, hospital records, and/or patient interviews; deaths were ascertained from death certificates, hospital records, and by linkage with the national vital status registry. A physician reviewed and coded all events using the International Classification of Diseases version 10 (ICD-10). Then, based on these codes, this physician identified and assessed cardiovascular events according to the Cardiovascular and Stroke Endpoint Definitions for Clinical Trials [25]. One senior cardiologist further adjudicated all cardiovascular (CV) deaths. The main study outcome was the occurrence of a four-point major adverse cardiovascular event composite (MACE), a composite criterion comprising cardiovascular death, stroke, myocardial infarction, or hospitalization for heart failure. We also assessed KRT initiation as a secondary outcome, defined by the initiation of dialysis or preemptive kidney transplantation, ascertained through patient or family interview, medical records, and linkage with the national REIN (French Renal Epidemiology and Information Network) registry of KRT [26]. Non-cardiovascular death and KRT initiation were competing events in the MACE analysis; all-cause death was a competing event in the analysis of KRT initiation. Patients were followed until initiation of KRT, death before KRT, completion of 5 years of follow-up, lost to follow-up, or censoring on 31 December 2020.

### Statistical analysis

Data are presented as median (IQR, interquartile range) for quantitative variables, and as counts and percentages for categorical variables. Median follow-up was estimated using the inverse Kaplan–Meier method [27].

The study was designed as a target trial emulation (Table S2). To test the effect of discontinuation versus continuation RAS inhibitors on MACE (time-to-event outcome with competing risks), we implemented the parametric g-formula to account for treatment-confounder feedback loop [28] (Fig. S1). Compared to the target clinical trial, this approach only allows estimation of a per-protocol effect of the intervention strategy. Our main and secondary outcomes were, respectively, the 3-year risk ratios of MACE and of KRT between the two hypothetical study interventions initiated at study baseline (i.e. static interventions). We depicted risk ratios of MACE and KRT between RAS inhibitor groups monthly until 3 years, and the associated simulated covariate histories. We assessed model fit by plotting the parametric and observed risk estimates and associated covariate histories under the natural course (actual treatment strategy).

The two primary steps of the parametric g-formula are (Step 1) modeling the joint distribution of all variables (Table S3) and (Step 2) simulating variables over the follow-up period using the estimates from Step 1. Overall, we aimed to account for potential time-fixed (age, sex, diabetes, history of heart failure, history of respiratory diseases, and uACR) and time-varying confounders (eGFR, systolic BP, serum sodium and potassium, diuretic prescriptions). These variables were chosen for their clinical relevance. In the analysis dataset, follow-up time was discretized at the monthly level. Information on time-dependent variables was considered from 3 months prior to baseline (with the objective to account for variable history) until event onset or censoring, with monthly updates. We used the last observation carried forward approach to deal with missing longitudinal data. As suggested by Hernan *et al*. [29, 30], we introduced information on the visit process in our models (i.e. a dummy variable indicating covariate measurement) to specify deterministic relations and potentially related time-varying confounding. Patients were censored at completion of the CKD-REIN follow-up by clinical research associates and, in a sensitivity analysis, at 12 months from the last covariate measurement. Other sensitivity analyses consisted of setting discontinuation status without any lag or with a lag of 3 months, changing g-formula parametrization (i.e. reversing the order of covariates with no obvious temporal order in Step 1), and excluding patients whose RAS inhibitor discontinuation could be linked to an adverse drug reaction.

The 95% confidence intervals for the risk ratios were was based on non-parametric bootstrap (2000 samples, 10 000 simulated patients per sample). Statistical analyses were performed using R, version 4.1.2 (R Foundation for Statistical Computing). In particular, g-formula was implemented with the gfoRmula [29] package for R, version 1.0.3.

## RESULTS

### Population

We identified 1604 (53%) patients who were prescribed a RAS inhibitor for at least 3 months while having an eGFR <30 ml/min/1.73 m^2^ (Fig. 1). After further excluding patients with missing covariate data and short follow-up periods (<1 month after meeting eligibility), 1434 (47%) patients fulfilled all inclusion criteria.

**Figure 1:**
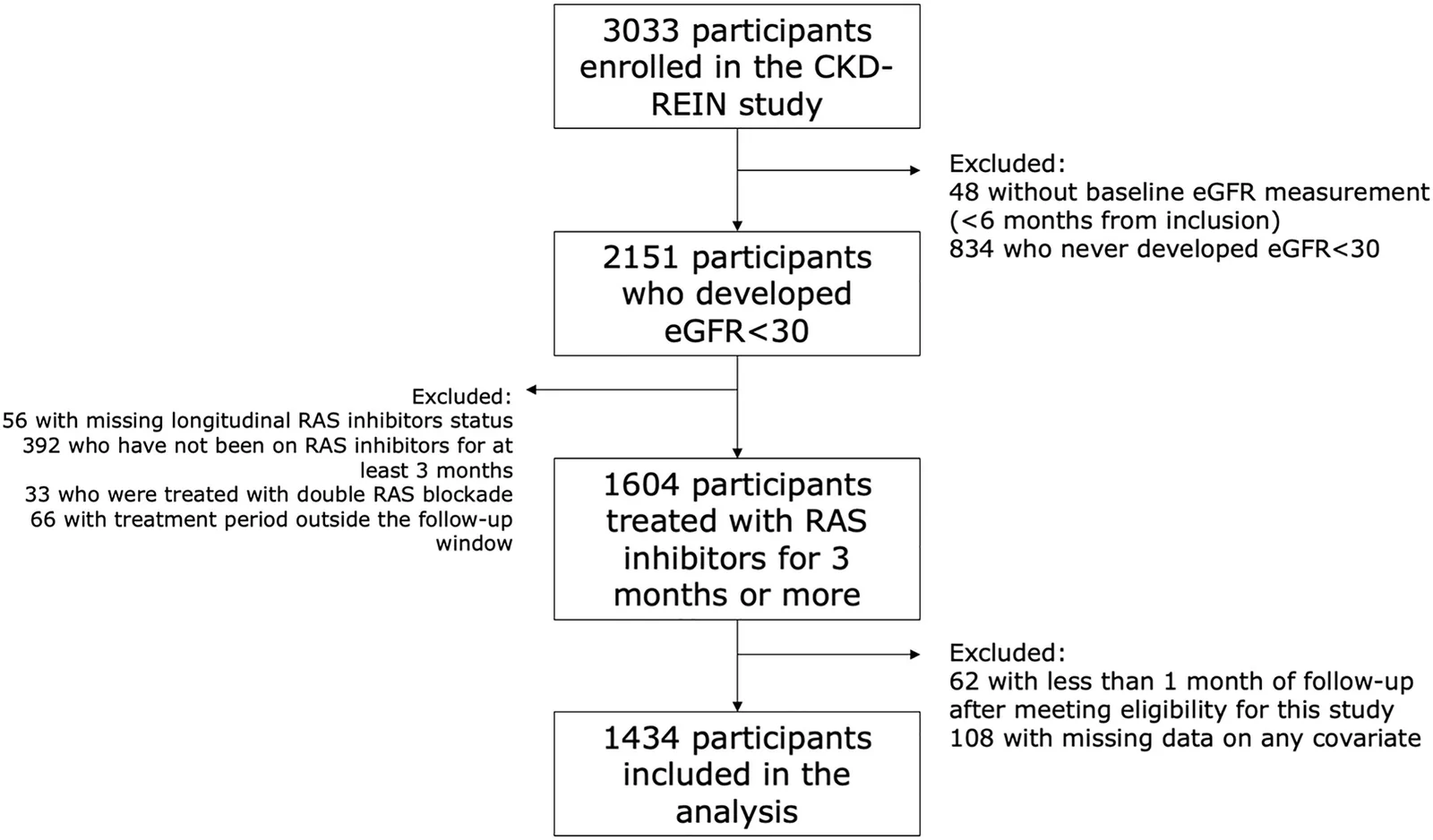
Flow chart of patient selection. Flow chart describing the selection of patients in the CKD-REIN cohort. A dual blockade involves the concomitant prescription of an ACE inhibitor and an angiotensin II receptor blocker.

In our population (Table 1), the median age was 68 years and 35% were women. At inclusion, these patients were mostly overweight (median BMI, 28 kg/m²), and only 35% had a high school diploma. The most common cause of nephropathy was diabetes (23%), followed by hypertension (22%) and glomerular nephropathy (19%). Most patients had dyslipidemia (79%), half had diabetes, a quarter had respiratory and/or coronary artery disease. The median BP was 140/78 mm Hg, and were prescribed a median of three antihypertensive drugs. Median eGFR was 25 ml/min/1.73 m^2^, albuminuria, 221 mg/g, and potassium, 4.6 mmol/l. ARBs accounted for most RAS inhibitor prescriptions (57%, Table S4); antihypertensive co-prescriptions were mainly diuretics (63%) and calcium channel blockers (51%).

**Table 1:** Patient baseline characteristics.

Characteristics	Overall (*n* = 1434)
General characteristics	
Age (years), median (IQR)	68 (59–76)
Women, *n* (%)	495 (35)
BMI (kg/m^2^), median (IQR)	28 (25–32)
Smoker or former smoker, *n* (%)	843 (60)
Education, *n* (%)	
Less than high school	930 (65)
High school	492 (35)
Kidney disease, *n* (%)	
Diabetes	332 (23)
Hypertensive	312 (22)
Glomerular	273 (19)
Tubulointerstitial	145 (10)
ADPKD	103 (7.2)
Vascular	84 (5.9)
Others	118 (8.2)
Unknown	67 (4.9)
Medical history, *n* (%)	
Hypertension	1362 (95)
Dyslipidemia	1128 (79)
Diabetes	664 (46)
Coronary artery disease	373 (26)
Respiratory diseases	360 (25)
Cancer	241 (17)
Peripheral arterial disease	254 (18)
Heart failure	178 (12)
Atrial Fibrillation	145 (10)
Cerebrovascular disease	100 (7.0)
Cirrhosis	24 (1.7)
Blood pressure parameters	
Systolic BP (mm Hg), median (IQR)	140 (130–154)
Diastolic BP (mm Hg), median (IQR)	78 (70–85)
Uncontrolled BP (BP ≥130/80 mm Hg), *n* (%)	1086 (76)
Uncontrolled BP (BP ≥140/90 mm Hg), *n* (%)	726 (51)
Number of antihypertensive drugs prescribed, median (IQR)	3 (2–4)
Laboratory	
eGFR (ml/min/1.73 m²), median (IQR)	25.5 (21.3–28.2)
Urine albumin-to-creatinine ratio (mg/g), median (IQR)	221 (39–677)
Sodium (mmol/l), median (IQR)	140 (139–142)
Potassium (mmol/l), median (IQR)	4.6 (4.3–4.9)
Calcium (mmol/l), median (IQR)	2.35 (2.27–2.43)

ADPKD, Autosomal Dominant Polycystic Kidney Disease.

During the study period, a total of 386 (27%) patients discontinued RAS inhibitors, with 426 discontinuation episodes (9.8/100 patient-years). The median (IQR) time from inclusion to discontinuation was 16 (6–29) months, and the eGFR at first discontinuation, 22 (16–27) ml/min/1.73 m² (Table S5). Patients with more comorbidities (except those with cancer), an eGFR of more than 30 ml/min/1.73 m² at enrollment in the original cohort (i.e. incident eGFR <30 ml/min/1.73 m² for the present analysis), or a lower level of education had higher rates of RAS inhibitor discontinuation (Fig. S2).

### Adverse drug reactions

Among our study population, over the entire study period, 70 relevant adverse drug reactions (ADRs) were identified, i.e. 1.6 ADRs per 100 patient-years (Table 2). This comprised 23 ADRs occurring in the 3 months immediately preceding RAS inhibitor discontinuation, i.e. 22 ADRs per 100 patient-years.

**Table 2:** Adverse drug reactions over the study period and in the 3 months immediately preceding RAS inhibitor discontinuation.

Type of event	Total number of ADRs in our population and over the study period (*n* = 70)	Incidence of ADRs in our population and over the study period (1.6 ADRs per 100 PY)	Number of ADRs in the 3 months preceding RAS inhibitor discontinuation (*n* = 23)	Incidence of ADRs in the 3 months preceding RASi discontinuation (22 ADRs per 100 PY)
Acute kidney injury, *n* (%)	51 (54%)	1.2 ADRs per 100PY	20 (63%)	18.8 ADRs per 100PY
Hypotension, *n* (%)	7 (7%)	0.2 ADR per 100PY	2 (6%)	1.9 ADRs per 100PY
Hyperkalemia, *n* (%)	12 (13%)	0.3 ADR per 100PY	1 (3%)	0.9 ADR per 100PY

PY, patient-years.

The most common ADR was AKI—1.2 ADR per 100 patient-years overall. The incidence rates of relevant ADRs were higher in the 3 months preceding RAS inhibitor discontinuation compared to the overall study period.

### Study outcomes

Over a median (IQR) follow-up of 35 (17–51) months, 228 patients had a MACE, 399 initiated KRT, and 144 died from a non-cardiovascular cause before KRT initiation.

Parametric g-formula simulations indicated that, at 3 years, eGFR was 1.25 ml/min/1.73 m^2^ higher and serum potassium, 0.25 mmol/l higher, in the continuation group (Fig. 2). Conversely, simulated serum sodium and systolic BP were higher (1 mmol/l and 2.5 mm Hg, respectively) in the discontinuation group. The simulated natural histories of RAS inhibitor prescription and study covariates in our population (Fig. S3) was very similar to their observed trends, non-parametrically estimated trends, except for prescriptions of loop diuretic and, to a lesser extent, that of thiazide diuretics. For these covariates, the gap between the parametric and the non-parametric estimates increased over time.

**Figure 2:**
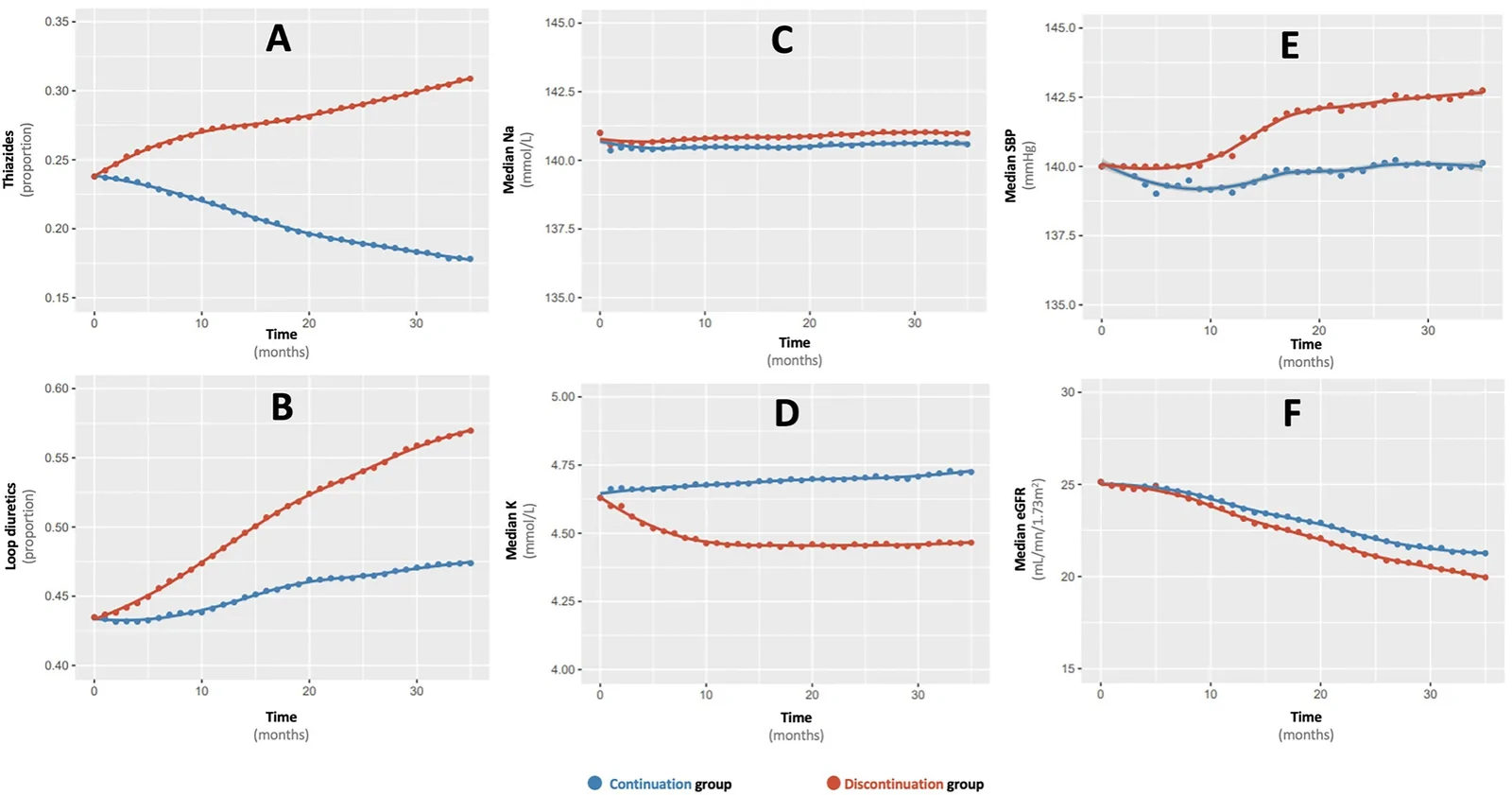
Parametric g-formula simulation of clinical and biological parameters over time the study period, by RAS inhibitor treatment group. The blue curve represents the continuation strategy and the red curve the discontinuation strategy. The different subgraphs correspond to: (A) thiazides—%, (B) loop diuretics—%, (C) median Na—mmol/l, (D) median K—mmol/l, (E) median SBP—mmHg, (F) median eGFR—mmol/l. The adjustment variables were: at baseline (time-fixed): age, sex, diabetes mellitus, chronic heart failure, respiratory disease; over follow-up (time-dependent): eGFR, systolic blood pressure, serum sodium and potassium, medication use (thiazide diuretic, loop diuretic). SBP, Systolic Blood Pressure.

The risk of MACE under discontinuation was twice as high as under continuation (Relative Risk (RR) 2.02, 95% CI 1.62–2.47, Fig. 3). The 3-year risk of KRT initiation was also higher in the discontinuation versus continuation simulated groups (RR 1.34, 95% CI 1.14–1.60).

**Figure 3:**
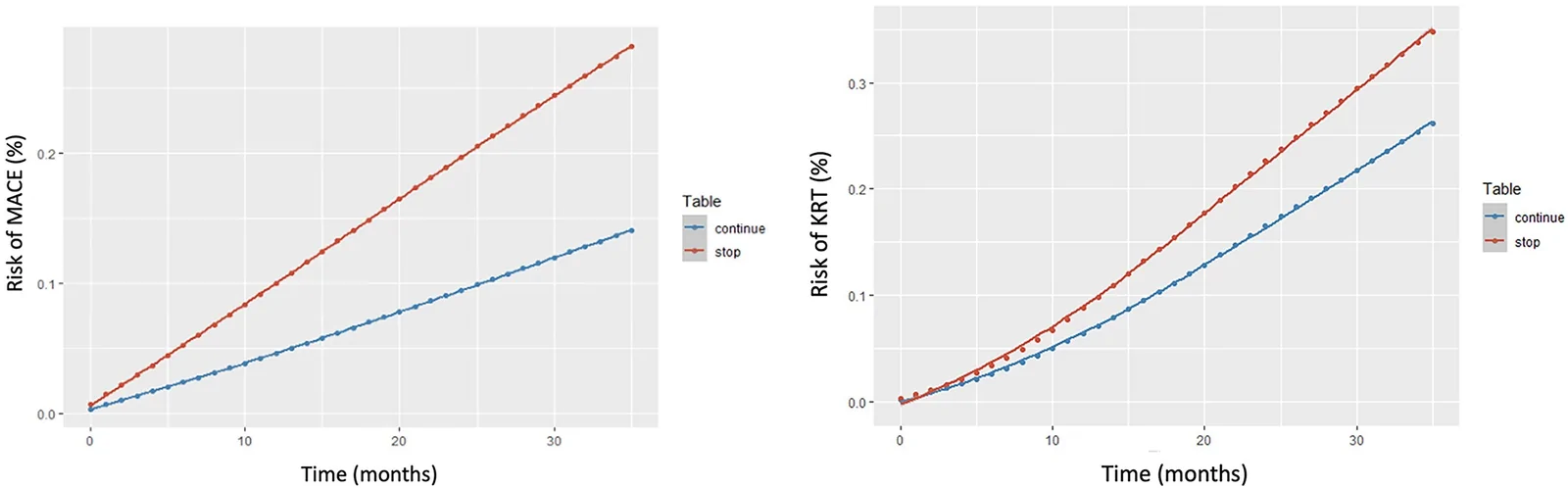
Parametric g-formula risk estimates of MACE (left) and KRT (right) over time the study period, by RAS inhibitor treatment group. The blue curve represents the discontinuation strategy and the red curve the continuation strategy.

#### Sensitivity analyses

Results were virtually unchanged when no time lag was considered to assign the discontinuation status, when the g-formula parametrization was changed, or when patients presenting relevant ADRs in the 3 months preceding RAS inhibitor discontinuation were excluded (Table S6). Assigning discontinuation status with an increased time lag than that used in the main analysis (3 months vs. 1 month, respectively), resulted in a lower RR of MACE of 1.80 (95% CI, 1.44–2.20). Censoring observations at 12 months from the last covariate measurement resulted in a higher RR of MACE of 2.13 (95% CI, 1.58–2.82).

## DISCUSSION

In our study based on patients with prevalent and incident advanced CKD, we found, after adjustment for potential confounders—including time-dependent ones—a two-fold higher risk of MACE and a 34% higher risk of KRT initiation after 3 years of RAS inhibitor discontinuation (vs. continuation). While the rate of RAS inhibitor discontinuation was relatively high, 9.8 per 100 patient-years, the rate of relevant ADRs was low, 1.6 per 100 patient-years. It is noteworthy, however, that the incidence rate of relevant ADRs in the 3-month period preceding RAS inhibitor discontinuation was almost 13 times that over the entire study period. Patients who discontinued RAS inhibitor had higher prevalence of diabetes and cardiovascular diseases at baseline than those who did not. Yet, patients with these health conditions are also those most likely to benefit from RAS inhibitor for secondary prevention. Our study adds to the current evidence by estimating the effect of sustained RAS inhibitor discontinuation versus continuation within a target trial framework incorporating repeated measures of kidney function, blood pressure, serum potassium, comorbidities, medication use, and ADRs. Compared with previous observational studies, our analysis incorporated a broader set of repeatedly assessed clinical and biological factors. Qiao *et al*. [31] relied mainly on baseline covariates (and no antihypertensive drugs), whereas albuminuria and serum potassium were not included in Yang *et al*. [12], and were missing in 54% and 37% of participants, respectively, in Fu *et al*. [9]. Our study therefore provides a more granular assessment of the clinical context surrounding RAS inhibitor discontinuation. This approach complements previous observational studies and the STOP-ACEi trial [8], which differed in their study populations, treatment strategies, follow-up durations, primary outcome definitions, and methodological approaches. In addition, the recruitment of patients from a nationally representative sample of nephrology real-life practices may help inform future recommendations.

We have summarized the findings of these four major studies [8, 9, 12, 31] (Table S7). All of these studies indicated an increase in the risk of MACE with hazard ratios of around 1.3. In terms of KRT, Yang *et al*. study [12] showed an increase in events after discontinuation, i.e. hazard ratio 1.30 (95% CI, 1.18–1.43), while the studies by Qiao *et al*. [31], Fu *et al*. [9], and Bhandari *et al*. [8] reported no risk difference. Our study population was generally similar to those of previous studies with respect to age and diabetes prevalence, with the exception of the study by Yang *et al*., in which all participants had diabetes. Mean systolic BP was around 140 mm Hg in most studies; instead, among patients in the United States, Qiao *et al*. reported a baseline systolic BP of 125 mm Hg, which reflects the more stringent BP targets in the United States [32] compared to Europe [6]. Notably, there were a number of major methodological differences across these studies: follow-up durations ranging from 12 to 60 months, use of various adjustment techniques (propensity score matching, weighting, conditional mixed linear regression), and heterogeneous definitions of MACE.

In patients with CKD and eGFR <60 ml/min/1.73 m^2^, the benefits of RAS inhibitors in terms of mortality, cardiovascular events, and CKD progression have been underlined in a large meta-analysis of 119 randomized controlled trials involving 64 768 patients. However, the level of evidence for their protective effect appeared to be weaker in patients with more advanced CKD—stages 4 and 5 [33, 34]. Uncertainty regarding the risk–benefit balance of RAS inhibitor in advanced CKD may partly explain heterogeneity in prescription practices and the lower frequency of RAS inhibitor prescription in patients with stages 4 and 5 CKD in the United States, compared to other countries [14, 15, 35].

Among the ADRs that may prompt clinicians to discontinue RAS inhibitors, acute kidney injury and worsening kidney function were the most frequently reported. Although the absolute number of ADRs was low, their incidence rates were higher during the 3-month period preceding RAS inhibitor discontinuation than during the overall follow-up, suggesting that these events frequently trigger treatment interruption. Our findings also emphasize that such discontinuation should not necessarily be considered permanent. Once the acute event has resolved and the benefit–risk balance has been reassessed, reintroduction of RAS inhibitors should be considered whenever clinically appropriate, as unnecessary long-term discontinuation may increase cardiovascular risk [36]. At the same time, the risk of acute-on-chronic kidney disease should not be underestimated, particularly in situations of physiological stress such as fever, vomiting, or hot weather, which warrant close clinical monitoring. In this context, patient education regarding self-management strategies, including “sick day rules,” may play an important supportive role [37]. A degree of selection bias among patients who discontinued RAS inhibitors cannot be excluded. However, adjustment for key biological parameters, including serum potassium and eGFR levels, is expected to partially account for this bias.

The number of hypotension and hyperkalemia episodes was low in our study. These hyperkalemia episodes may be due to redistribution via metabolic acidosis with increased anion gap rather than urine depletion [38]. The hyperkalemia rate in the Yang *et al*. study [12] was 9.98 per 100 patient-years overall, 10.1 and 9.96 per 100 patient-years in the discontinuation and continuation groups, respectively. In STOP-ACEi [8], there were 2 hyperkalemia episodes in the discontinuation group versus 4 in the continuation group. This probably means that these events are preventable if patients are well monitored. Moreover, the strategy of discontinuing RAS inhibitors after a first episode of hyperkalemia (serum potassium >5 mmol/l) may be associated with an increased risk of MACE and mortality [39]. The availability of new potassium-binding agents [40], or new kidney- and cardioprotective agents that are less hyperkalemic, such as finerenone [41], further strengthens the case for continued RAS inhibitor in advanced CKD patients.

### Strengths and limitations

Our study has several strengths. It is based on a large number of patients with confirmed CKD recruited from a nationally representative sample of nephrology centers. Our prospective data collection, in particular, with a detailed ascertainment of prescribed medications, ADRs, and cardiovascular and kidney outcomes, allowed us to take important time-varying confounders into account and to limit loss to follow-up. The target trial emulation using the parametric g-formula was particularly suited to this question, as it accounted for time-varying confounders affected by prior RAS inhibitor exposure and estimated the effect of sustained discontinuation versus continuation strategies. This approach appropriately addressed treatment-confounder feedback and limited biases inherent to conventional survival analyses, including immortal time bias. It also enabled estimation of the trajectories of key time-varying clinical variables under each strategy, such as eGFR, systolic blood pressure, and serum potassium, facilitating interpretation of the estimated treatment effects [19].

This analysis also has limitations. We performed complete case analysis assuming a missing completely at random (MCAR)—mechanism for missing covariate data. While MCAR is a strong, unverifiable assumption, the percentage of missing covariate data was limited (7% of eligible participants) and no substantial difference was observed between the studied population and participants excluded due to missing covariate data (Table S8). Information on antihypertensive drugs was obtained from prescription records, so we were unable to verify actual drug dispensing or use. Because CKD-REIN was initiated before the widespread use of sodium–glucose cotransporter 2 inhibitors (SGLT2i) and angiotensin receptor–neprilysin inhibitors (ARNIs), no participant was receiving an SGLT2i and only 15 were receiving an ARNI at inclusion, which may limit the generalizability of our findings to contemporary clinical practice. Nevertheless, RAS inhibitors remain a cornerstone of CKD management and, as inexpensive generic medications, continue to be widely used worldwide. In addition, we were unable to assess drug doses. Although ADR collection was a central aspect of data collection in the CKD-REIN study, we may have missed some events—especially out-of-hospital ones—that might not have been reported by physicians in medical records or by participants to physicians or during interviews. Although the parametric g-formula can more efficiently deal with treatment confounder feedback compared to propensity score weighting, it relies on the assumption of correct specification of the exposure, time-varying confounders, and outcomes at each time point [42, 43]. Similarity between observed and parametrically estimated natural history does not indicate important model misspecification under treatment strategies, except perhaps for loop diuretics. Loop diuretic prescriptions are highly dynamic in advanced CKD, which may explain gaps between observed and simulated histories. While the impact of this inconsistency is uncertain, numerous sensitivity analyses support the robustness of our results. Finally, our analyses rely on the assumption that all relevant confounders for the treatment strategy–outcome relationship are measured and taken into account. In this study, we had updated information on the most important factors influencing RAS inhibitor prescription, e.g. eGFR level, systolic BP, serum potassium. ADRs, which could be a reason for discontinuing RAS inhibitor and a marker of outcome susceptibility, were rare and unlikely to influence our results.

## CONCLUSION

Compared to continuing RAS inhibitor in patients with advanced CKD, RAS inhibitor discontinuation was associated with a two-fold higher risk of MACE and a 34% higher risk of KRT initiation at 3 years. Our results suggest that cardiovascular and kidney protective benefits of RAS inhibitor therapy persist despite low kidney function. ADRs, particularly acute kidney injury, occurred more frequently in the period immediately preceding treatment discontinuation, indicating that such events likely informed clinical decisions to discontinue therapy. Strategies to improve patient monitoring and self-management, particularly in the context of high-risk scenarios, together with the use of newer potassium binders to facilitate RAS inhibitor continuation in patients with hyperkalemia, could optimize the balance between therapeutic benefits and risks, paving the way for more individualized treatment protocols in advanced CKD management.

**LIST OF ABBREVIATIONS**
ACEi: Angiotensin-Converting Enzyme inhibitors
ADRs: Adverse Drug Reactions
AKI: Acute Kidney Injury
ARBs: Angiotensin II Receptor Blockers
BMI: Body Mass Index
BP: Blood pressure
CKD: Chronic Kidney Disease
CKD-EPI: Chronic Kidney Disease Epidemiology Collaboration
CKD-REIN: CKD-Renal Epidemiology and Information Network
eGFR: estimated Glomerular Filtration Rate
HbA1c: Glycated Hemoglobin
IQR: Interquartile Range

## Supplementary Material

sfag304_Supplemental_File

## Data Availability

The data presented in this study are available on request from N.A.P. The data are not publicly available due to confidential reasons of the national CKD-REIN study.
