# Supplementary material for: Impact of renin–angiotensin system inhibitors discontinuation in advanced chronic kidney disease: a target trial emulation
Source: Clin Kidney J. 2026 Sep 4;19(10):sfag304. doi: 10.1093/ckj/sfag304 (PMC13645658; doi:10.1093/ckj/sfag304)
Supplement: Supplementary file 1 [file sfag304_supplemental_file.docx]

**Impact of renin-angiotensin system inhibitors discontinuation in advanced chronic kidney disease: a target trial emulation**

Valentin Maisons^1,2^, Margaux Costes-Albrespic^3^, Sophie Liabeuf^4,5^, Solène M. Laville^4,5^, Ziad Massy^3,6^, Angelo Karaboyas^7^, Roberto Pecoits‐Filho^7^, Emilie Moutard^3^, Marie Metzger^3^ Jean-Michel Halimi^1^, Etienne Dantan^2^, Natalia Alencar de Pinho^3,#^, Bénédicte Sautenet^1,2,#^

**SUPPLEMENTARY DATA**

**Address correspondence to:**

Valentin Maisons, MD, Nephrology,
Department of Nephrology, CHU Bretonneau, 2 boulevard Tonnellé, 37000 TOURS, France.
[Valentin.maisons@univ-tours.fr](mailto:Valentin.maisons@univ-tours.fr)
+332 47 47 37 46

**Running headline:** Discontinuation of renin-angiotensin system inhibitors in advanced chronic kidney disease

**Legends to supplemental figures**

**Supplementary Figure 1.** Directed acyclic graph depicting treatment-confounder feedback loop in the evaluation of the effect of RAS inhibitor discontinuation in an observation study.

Directed acyclic graph (DAG) illustrating the treatment-confounder feedback loop in the evaluation of the effect of renin-angiotensin system (RAS) inhibitor discontinuation in an observational study. Arrows represent causal pathways, highlighting the complex interplay between confounders, treatment decisions, and outcomes. The diagram emphasizes the importance of accounting for confounding factors to ensure valid inference.

*Abbreviations: BP, Blood Pressure; eGFR, Estimated Glomerular Filtration Rate; KRT, Kidney Replacement Therapy; MACE, Major Adverse Cardiovascular Events; RAS, Renin-Angiotensin System.*

**Supplementary Figure 2.** Incidence of RAS inhibitors discontinuation by baseline characteristics.

Incidence of RAS inhibitors discontinuation stratified by baseline characteristics. For each covariate, the figure displays a point with 95%-CI-bar representing the incidence when the condition is met (covariate = 1) and a red bar representing the incidence when the condition is not met (covariate = 0). This highlights the variation in discontinuation rates based on different patient subgroups.

Blood pressure (BP) control status is categorized as: uncontrolled BP (A) if BP ≥ 130/80 mm Hg and uncontrolled BP (B) if BP ≥ 140/90 mm Hg.

*Abbreviations: BP, Blood Pressure; eGFR, Estimated Glomerular Filtration Rate.*

**Supplementary Figure 3.** Parametric and observed risk estimates and covariates history under the natural course.

The blue solid lines represent parametric estimates derived from the fitted g-formula model, while the red solid lines correspond to observed (non-parametric) estimates. Covariate history and risk estimates are displayed over time, illustrating the model's ability to capture temporal trends in the absence of intervention.

*Abbreviations: SBP, Systolic Blood Pressure; eGFR, Estimated Glomerular Filtration Rate; mes eGFR, estimated glomerular filtration rate measurement; mes electro, electrolyte measurement; mes SBP, Systolic Blood measurement; RAS inhibitor, renin-angiotensin system inhibitor.*

**Supplementary Figure 1.** Directed acyclic graph depicting treatment-confounder feedback loop in the evaluation of the effect of renin-angiotensin system inhibitor discontinuation in an observation study.

**Supplementary Figure 2.** Incidence of RAS inhibitors discontinuation by baseline binary characteristics.

**Supplementary Figure 3.** Parametric and observed risk estimates and covariates history under the natural course

**Supplementary Table 1.** Anatomical Therapeutic and Chemical (ATC) codes of antihypertensive drugs

| Antihypertensive drugs | **ATC codes** |
| --- | --- |
| Thiazide diuretics | C02LA01 - C03AA03 - C03BA11 - C03BX03 - C03EA01- C07BB02 - C07BB07 - C07BB12 - C07CB03 - C07DA06 - C08GA02 - C09BA01 - C09BA02 - C09BA03 - C09BA04 - C09BA05 - C09BA06 - C09BA07 - C09BA09 - C09BA15 - C09BX01 - C09DA01 - C09DA03 - C09DA04 - C09DA06 - C09DA07 - C09DA08 - C09DX01 - C09EA04 - C09XA52 |
| High-ceiling diuretics | C03CA01 - C03CA02 - C03CA03 - C03EB01 |
| Mineralocorticoid receptor blockers | C03DA - C03EA04 |
| Amiloride | C03DB01 - C03EB01 - C07DA06 - C03EA01 |
| Beta-blockers | C07AA03 - C07AA05 - C07AA06 - C07AA12 - C07AA16 - C07AB02 - C07AB03 - C07AB04 - C07AB05 - C07AB07 - C07AB08 - C07AB12 - C07AG01 - C07BB02 - C07BB07 - C07BB12 - C07CB03 - C07DA06 - C07FB02 - C07FB03 - C09BX02 |
| Calcium channel blockers | C08CA01 - C08CA02 - C08CA03 - C08CA04 - C08CA05 - C08CA08 - C08CA09 - C08CA11 - C08CA13 - C08DA01 - C08DB01 - C08GA02 - C07FB02 - C07FB03 - C09DX01 - C09BB02 - C09BB04 – C09BB07 - C09BB10 - C09DB01 - C09DB02 - C09DB04 - C09DB05 - C09BX01 - C10BX03 |
| ACE inhibitors | C09AA01 - C09AA02 - C09AA03 - C09AA04 - C09AA05 - C09AA06 - C09AA07 - C09AA08 - C09AA09 - C09AA10 - C09AA15 - C09BB02 - C09BB04 - C09BB07 - C09BB10 - C09BA01 - C09BA02 - C09BA03 - C09BA04 - C09BA05 - C09BA06 - C09BA07 - C09BA09 - C09BA15 - C09BX01 - C09BX02 |
| Angiotensin II antagonists | C09CA01 - C09CA02 - C09CA03 - C09CA04 - C09CA06 - C09CA07 - C09CA08 - C09DA01 - C09DA03 - C09DA04 - C09DA06 - C09DA07 - C09DA08 - C09DB01 - C09DB02 - C09DB04 - C09DB05 - C09DX01 – C09DX04 |
| Renin-inhibitors | C09XA02 - C09XA52 |
| Methyldopa | C02AB02 |
| Imidazoline receptor agonists | C02AC01 - C02AC05 - C02AC06 |
| Alpha-adrenoreceptor antagonists | C02CA01 - C02CA04 - C02CA06 |
| Pyrimidine derivatives | C02DC01 |

**Supplementary Table 2.** Study protocol of the target trial emulation.

| Characteristics | Target Trial | Emulation with Observational Data from CKD-Rein |
| --- | --- | --- |
| Eligibility criteria | Individuals 18 year or older, under nephrologist care with CKD 4 or 5 (i.e., eGFR < 30ml/min per 1.73m^2^), no history of kidney transplantation or dialysis, taking RAS inhibitor for at least 3 months, without double blockade of the renin angiotensin system. | Same as target trial  Operationally, we selected patients from the CKD-REIN cohort study who were either included with or developed eGFR < 30 ml/min/1.73m². They also had to be prescribed RAS inhibitor for at least 3 months during the observation period. |
| Treatment strategies | Discontinuation or continuation of RAS inhibitors at study baseline | Same as target trial |
| Treatment assignment | Randomization, no blinding | Eligible individuals were assigned to a treatment strategy in a monthly basis, according to whether or not they discontinued RAS inhibitors for more than one month. To account for confounding that may arise from non-randomized allocation to study groups, we used the G-formula to deal with the following time-fixed and time-varying confounding factors:  - at baseline (time-fixed): age, sex, diabetes mellitus, chronic heart failure, respiratory disease, urinary albumin-to-creatinine ratio  - over follow-up (time-dependent): eGFR, systolic blood pressure, serum sodium and potassium, medication use (thiazide diuretic, loop diuretic). |
| Outcomes | 1. MACE- 4 points : cardiovascular death, stroke, myocardial infarction or hospitalization for heart failure 2. Kidney replacement therapy (Transplantation or dialysis) | Same as the target trial. |
| Causal estimand | Intention-to-treat effect (study groups based on randomization)  Per protocol effect (study groups based on whether participants received the treatment strategy specified in protocol) | Per protocol, average treatment effect in the entire study population (ATE) |
| Start and end of follow-up | Starts at randomization and ends at occurrence of the end point, administrative censoring or at 3 years of follow-up | Starts 3 months after patients have met operational criteria for inclusion (eGFR < 30 ml/min/1.73m² and at least 3 months of observed RAS inhibitor prescription); ends at the occurrence of the end point, censoring or at 3 years of follow-up |
| Statistical analysis | Cumulative incidence functions to estimate event probabilities across study groups  and Cox-proportional hazard models to estimate cause-specific hazard ratios and their 95% confidence interval | Parametric G-formula to estimate the risk ratio between the two interventions (to discontinue Vs. to continue RAS inhibitors) at 3 years, along with its 95% confidence interval based on percentiles of a bootstrap distribution (2,000 samples; 10,000 simulated patients per sample). We further extracted from the G-formula outputs: the risk of the study outcomes and the simulated histories from step 2 of the algorithm under the specified interventions at each follow-up time point. |

*Abbreviations: eGFR, Estimated Glomerular Filtration Rate; RAS, renin-angiotensin system.*

**Supplementary Table 3.** Parametrization used to model the joint distribution of time-dependent variables and outcomes.

| Dependent variable | Functional form of the dependent variable | Model independent variables |
| --- | --- | --- |
| eGFR measurement | Binary | age + sex + diabetes + hf + resp_dis + LogProt + 1ag1_egfr + lag_cumavg3_egfr + 1agl_SBP + lagl_k + lagl_na + lagl_thiaz_diur + lagl_loop_diur + lagl_RAS inhibitor + lag1_mes_egfr |
| eGFR | Bounded normal | age + sex + diabetes + hf + resp_dis + LogProt + 1ag1_egfr + lagl_SBP + lagl_k + lagl_na + lagl_thiaz_diur + lagl_loop_diur + lagl_RAS inhibitor + mes_egfr + to_f |
| Systolic blood pressure measurement | Binary | age + sex + diabetes + hf + resp_dis + LogProt + egfr + lag_cumavg3_egfr + lagl_SBP + lagl_k + lagl_na + lagl_thiaz_diur + lagl_loop_diur + lagl_RAS inhibitor + mes_egfr |
| Systolic blood pressure | Normal | age + sex + diabetes + hf + resp_dis + LogProt + egfr + lagl SBP + lagl_k + lag_na + lagl_thiaz_diur + lag_loop_diur + lagl_RAS inhibitor + mes_SBP |
| Ionogram measurement | Binary | age + sex + diabetes + atcd ic + resp_dis + LogProt + dfa + SBP + lagl_loop_diur + lagl_RAS inhibitor + mes_egfr |
| Kaliemia | Bounded normal | age + sex + diabetes + hf + resp_dis + LogProt + egfr + SBP + lagl_k + lagl_na + lagl_thiaz_diur + lagl_loop_diur + lagl_RAS inhibitor + mes_nak + to_f |
| Natremia | Bounded normal | age + sex + diabetes + hf + resp_dis + LogProt + egfr + SBP + k + lagl_na + lagl_thiaz_diur + lagl_loop_diur + lagl_RAS inhibitor + mes_nak + to_f |
| Loop diuretic | Binary | age + sex + diabetes + hf + resp_dis + LogProt + egfr + SBP + k + na + lagl_thiaz_diur + lagl_loop_diur + lagl_RAS inhibitor + to_f |
| Thiazide | Binary | age + sex + diabetes + hf + resp_dis + LogProt egfr + SBP + k + na + lagl_thiaz_diur + loop_diur + lagl_RAS inhibitor + to_f |
| RAS inhibitors | Binary | age + sex + diabetes + hf + resp_dis + LogProt + egfr + SBP + k + na + thiaz_diur + loop_diur + lagl_RAS inhibitor + to_f |
| Outcome | Binary | tO + I(tO^2) + age + sex + diabetes + hf + resp_dis + LogProt + RAS inhibitor + thiaz_diur + loop_diur + egfr + SBP + na + k |

*Legend: The table presents the outcome (dependent) variables and their functional forms alongside the independent variables for each model. Outcome variables are noted with their specific functional forms, which can be either binary (0/1) or continuous (bounded normal or normal distributions). Independent variables in the models are characterized by their functional forms: continuous variables include : age, log-transformed urinary albumin-to-creatinine ratio (LogProt), egfr (estimated glomerular filtration rate), sbp (systolic blood pressure), k (kaliemia), and na (natremia) ; while binary variables include sex, diabetes, heart failure (HF), respiratory disease (resp_dis), loop diuretic (loop_diur), thiazide (thiaz_diur), and RAS inhibitors (RAS inhibitor). For lagged covariates, Lag_1 refers to the value of the covariate at the previous month (t-1), and lag_cumavg3 denotes the cumulative average of the covariate values from three prior months (t-3 through t-1). Prefixes in variable names denote specific details: "mes_" indicates a measurement taken at the current time point.*

*Abbreviations: eGFR, estimated glomerular filtration rate; HF, heart failure; Loop_diur, Lood diuretics; RAS inhibitor, Renin-angiotensin system inhibitors; Resp_dis, Respiratory disease.*

**Supplementary Table 4.** Antihypertensive and metabolic drugs classes prescribed at baseline, overall and by CKD-stage.

| Antihypertensive Drug classes | All  (n=1,434) | Stage 4  (n=1,371) | Stage 5  (n=63) |
| --- | --- | --- | --- |
| RAS inhibitors, n (%) | 1434 (100) | 1371 (100) | 63 (100) |
| ACE inhibitors | 608 (42) | 583 (43) | 25 (40) |
| Angiotensin II antagonists | 822 (57) | 785 (57) | 37 (59) |
| Direct renin inhibitors* | 4 (0.3) | 3 (0.2) | 1(1.6) |
| Diuretics, n (%) | 898 (63) | 857 (63) | 41 (65) |
| Thiazide and thiazide-like | 335 (23) | 324 (24) | 11 (18) |
| High-ceiling (loop) diuretics | 618 (43) | 586 (43) | 32 (51) |
| Mineralocorticoid receptor blockers | 50 (3.5) | 49 (3.6) | 1 (1.6) |
| Amiloride | 4 (0.3) | 4 (0.3) | 0 (0) |
| Calcium channel blockers, n (%) | 729 (51) | 692 (51) | 37 (59) |
| Beta-blocking agents, n (%) | 626 (44) | 593 (43) | 33(53) |
| Alpha-adrenergic antagonists, n (%) | 180 (13) | 164 (12) | 16 (25) |
| Centrally acting drugs, n (%) | 121 (8.5) | 115 (8.4) | 6 (9.5) |
| Pyrimidine derivatives, n (%) | 1 (0.1) | 1 (0.1) | 0 (0) |

Chronic Kidney disease G-stages are defined by the estimated glomerular filtration (eGFR) level or the need of Kidney replacement therapy (KRT): 4, 15≤eGFR<30 mL/min/1.73m²; and 5, eGFR<15 mL/min/1.73m² or KRT.

*Although direct renin inhibitors belong to the renin-angiotensin system inhibitor class, they are not recommended for first-line treatment of hypertension in CKD. They are no longer commercialized in France since October 2017.

Abbreviations: ACE, angiotensin converting enzyme; RAS, Renin-Angiotensin System

**Supplementary Table 5.** Patient characteristics at first renin-angiotensin system inhibitor discontinuation.

| Characteristics | Overall  (n=386) |
| --- | --- |
| Type of RAS inhibitor, n (%) |  |
| ACE inhibitors | 217 (56) |
| Angiotensin II antagonists | 169 (44) |
| General characteristics |  |
| Time between inclusion and RAS inhibitor discontinuation (months), median (IQR) | 16 [6-29] |
| Age (years), median [IQR] | 70 [62-77] |
| Women, n (%) | 138 (36) |
| Medical history |  |
| Diabetes, n (%) | 195 (51) |
| Respiratory diseases, n (%) | 120 (31) |
| Heart failure, n (%) | 57 (15) |
| Laboratory and clinical data |  |
| eGFR (mL/min/1.73m²), median [IQR] | 22 [16-27] |
| Sodium (mmol/L), median [IQR] | 141 [139-143] |
| Potassium (mmol/L), median [IQR] | 4.6 [4.2-5.0] |
| Systolic BP (mmHg), median [IQR] | 140 [129-154] |
| Albumin-to-creatinine ratio (mg/g), median [IQR] | 226 [41-668] |
| Associated antihypertensive drugs |  |
| Thiazide and thiazide-like, n (%) | 26 (6.7) |
| High-ceiling (loop) diuretics, n (%) | 201 (52) |
| Total number of antihypertensive drugs, n (%)  1  2  3  4  5 | 91 (24)  157 (41)  89 (23)  40 (10)  9 (2.3) |

Abbreviations: ACE, angiotensin converting enzyme; *BP, blood pressure*; *eGFR, estimated glomerular filtration rate; IQR, interquartile range*; RAS inhibitor, Renin-Angiotensin System inhibitor

**Supplementary Table 6.** Sensitivity analyses: G-formula relative risk estimates and 95% CI of MACE and KRT associated with renin-angiotensin system inhibitor discontinuation (versus continuation), according to different model parametrization.

| Type of analysis | MACE | KRT |
| --- | --- | --- |
| Discontinuation effect in the month of RAS inhibitor discontinuation | 2.01 [1.59-2.47] | 1.27 [1.07-1.50] |
| Discontinuation effect after 3 months of RAS inhibitor discontinuation | 1.80 [1.44-2.20] | 1.30 [1.10-1.53] |
| Reversing the order of loop and thiazide diuretics in covariate simulation | 2.01 [1.62-2.46] | 1.34 [1.14-1.59] |
| Reversing the order of blood pressure and eGFR (and related dummy measurement covariates) in covariate simulation | 2.01 [1.62-2.46] | 1.35 [1.14-1.58] |
| Censoring at 12 months from the last covariate measurement | 2.13 [1.58-2.82] | 1.44 [1.16-1.75] |
| Exclusion of patients whose discontinuation seems to be linked to a side effect | 2.00 [1.48-2.50] | 1.30 [1.11-1.54] |

Abbreviations: CI, Confidence Interval; eGFR, estimated glomerular filtration rate; KRT, Kidney Replacement Therapy; MACE, Major Adverse Cardiovascular Events; RAS inhibitor, Renin-Angiotensin System Inhibitors

**Supplementary Table 7.** Studies on the effect of discontinuation of RAS inhibitors in advanced chronic kidney disease.

| Study | Overall | Group | Sample size | Follow-up (Months) | Mean Age (Years) | Male (%) | Diab. (%) | Mean Systolic BP (mmHg) | Methodology | Primary Outcome | Meas. of the effect | MACE | Death | KRT |
| --- | --- | --- | --- | --- | --- | --- | --- | --- | --- | --- | --- | --- | --- | --- |
| ^8^  Bhandari 2022 | Randomized Controlled Trial | Continued Discontinued | 205 206 | 36 | 63  overall | 69 vs  68 | 38 vs  36 | 138 vs  136 | Mixed linear regression | eGFR at 3 years | HR | 1.22  [1.00-1.49]* | 0.90  [0.51-1.59] | 1.28  [0.99-1.65] |
| ^12^  Yang 2023 | Prospective cohort | Continued Discontinued | 8634 1766 | 12 | 73.3  vs 73.3 | 48.2 vs 48.2 | 100 vs 100 | 141 vs 141 | Propensity score overlap-weighting  Cox model | Death, MACE, HF, KRT | HR | 1.27  [1.08-1.49]* | 0.93  [0.86-1.01] | 1.30  [1.17-1.43]* |
| ^9^  Fu  2021 | Retrospective cohort | Continued Discontinued | ~8000 ~8000 * | 60 | 72  overall | 64.3  overall | 49.5 overall | 139 overall | Cloning, censoring, weighting, target trial emulation. Logistic regression. | Death (5years), MACE, KRT | Δ RMST diff. ** | -3.3 [-5.3 ; -1.4]* | -3.6 [-5.4 ; -1.8]* | 0.8 [-0.8 ; 2.5] |
| ^31^  Qiao 2020 | Retrospective cohort | Continued Discontinued | 1205 1205 | 60 | 73.3  vs 73.1 | 41.4 vs 42.2 | 48.9 vs 47.5 | 125.4 vs 125.3 | Propensity score matching  Cox model | eGFR at 5 years | HR | 1.37  [1.20-1.56]* | 1.39  [1.20-1.60]* | 1.19  [0.86-1.65] |

Abbreviations: BP, Blood Pressure; eGFR, estimated glomerular filtration rate; HF, Heart Failure; KRT, Kidney Replacement Therapy; MACE, Major Adverse Cardiovascular Events; NR, Not Reported; RAS inhibitor, Renin-Angiotensin System inhibitors
* Depending on the chosen outcome.
** ΔRMST diff : represents the mean difference in event-free survival time (months) between groups over the study period.

**Supplementary Table S8**. G-formula covariates of participants excluded or included based on the presence of missing covariate data.

| Characteristics | Excluded  (n=108) | Included  (n=1,434) |
| --- | --- | --- |
| Age (years), mean (SD) | 68 (14) | 67 (13) |
| Women | 64% | 65% |
| Diabetes | 50% | 46% |
| Respiratory diseases | 27% | 25% |
| Heart failure | 14% | 12% |
| Systolic BP (mm Hg), mean (SD) | 142 (21) | 142 (20) |
| eGFR (mL/min/1.73m²), mean (SD) | 22 (5.9) | 24 (6.0) |
| Log-urine albumin-to-creatinine ratio (mg/g), mean (SD) | 5.3 (2.0) | 5.1 (1.9) |
| Sodium (mmol/L), mean (SD) | 140 (2.7) | 140 (3.0) |
| Potassium (mmol/L), mean (SD) | 4.7 (0.51) | 4.7 (0.52) |
| Loop diuretic | 45% | 43% |
| Thiazide diuretic | 17% | 23% |

*Abbreviations: BP, blood pressure; eGFR, estimated glomerular filtration rate; SD, standard deviation*
